# A quantitative comparison of West Nile virus incidence from 2013 to 2018 in Emilia-Romagna, Italy

**DOI:** 10.1371/journal.pntd.0007953

**Published:** 2020-01-02

**Authors:** Giovanni Marini, Mattia Calzolari, Paola Angelini, Romeo Bellini, Silvia Bellini, Luca Bolzoni, Deborah Torri, Francesco Defilippo, Ilaria Dorigatti, Birgit Nikolay, Andrea Pugliese, Roberto Rosà, Marco Tamba

**Affiliations:** 1 Department of Biodiversity and Molecular Ecology, Research and Innovation Centre, Fondazione Edmund Mach, San Michele all’Adige (TN), Italy; 2 Epilab-JRU, FEM-FBK Joint Research Unit, Province of Trento, Italy; 3 Laboratory of Entomology, Istituto Zooprofilattico Sperimentale della Lombardia e dell’Emilia Romagna “B. Ubertini”, Reggio Emilia, Italy; 4 Public Health Service, Emilia-Romagna Region, Bologna, Italy; 5 Dept. Medical & Veterinary Entomology, Centro Agricoltura Ambiente “G. Nicoli”, Crevalcore, Italy; 6 Epidemiology Unit, Istituto Zooprofilattico Sperimentale della Lombardia e dell’Emilia Romagna “B. Ubertini”, Bologna, Italy; 7 Risk Analysis and Genomic Epidemiology Unit, Istituto Zooprofilattico Sperimentale della Lombardia e dell’Emilia Romagna “B. Ubertini”, Parma, Italy; 8 MRC Centre for Global Infectious Disease Analysis, Department of Infectious Disease Epidemiology, Imperial College London, London, United Kingdom; 9 Mathematical Modelling of Infectious Diseases Unit, Institut Pasteur, Paris, France; 10 CNRS UMR2000: Génomique évolutive, modélisation et santé, Institut Pasteur, Paris, France; 11 Center of Bioinformatics, Biostatistics and Integrative Biology, Institut Pasteur, Paris, France; 12 Department of Mathematics, University of Trento, Trento, Italy; 13 Center Agriculture Food Environment, University of Trento, San Michele all’Adige (TN), Italy; Saudi Ministry of Health, SAUDI ARABIA

## Abstract

**Background:**

West Nile virus (WNV) transmission was much greater in 2018 than in previous seasons in Europe. Focusing on Emilia-Romagna region (northern Italy), we analyzed detailed entomological and epidemiological data collected in 2013–2018 to quantitatively assess environmental drivers of transmission and explore hypotheses to better understand why the 2018 epidemiological season was substantially different than the previous seasons. In particular, in 2018 WNV was detected at least two weeks before the observed circulation in 2013–2017 and in a larger number of mosquito pools. Transmission resulted in 100 neuroinvasive human cases in the region, more than the total number of cases recorded between 2013 and 2017.

**Methodology:**

We used temperature-driven mathematical models calibrated through a Bayesian approach to simulate mosquito population dynamics and WNV infection rates in the avian population. We then estimated the human transmission risk as the probability, for a person living in the study area, of being bitten by an infectious mosquito in a given week. Finally, we translated such risk into reported WNV human infections.

**Principal findings:**

The estimated prevalence of WNV in the mosquito and avian populations were significantly higher in 2018 with respect to 2013–2017 seasons, especially in the eastern part of the region. Furthermore, peak avian prevalence was estimated to have occurred earlier, corresponding to a steeper decline towards the end of summer. The high mosquito prevalence resulted in a much greater predicted risk for human transmission in 2018, which was estimated to be up to eight times higher than previous seasons. We hypothesized, on the basis of our modelling results, that such greater WNV circulation might be partially explained by exceptionally high spring temperatures, which have likely helped to amplify WNV transmission at the beginning of the 2018 season.

## Author summary

West Nile virus (WNV) is one of the most recent emerging mosquito-borne diseases in Europe and North America. While most human infections are asymptomatic, about 1% of them can result in severe neurological diseases which might be fatal. WNV transmission was unusually greater in 2018 than in previous years in many European countries, resulting in a large number of human infections. Focusing on Emilia-Romagna region (Italy), we developed an epidemiological model informed by entomological data; through that we found that exceptionally high spring temperatures might have contributed at amplifying WNV transmission at the beginning of the season, causing greater WNV prevalence in mosquito and avian populations during the summer, which resulted in a higher estimated risk for human transmission. Thus, weather anomalies at the beginning of the mosquito breeding season, which are likely to become more common under the projected scenarios of climate change, might act as an early warning signal for public health authorities, enabling them to design efficient surveillance and prevention strategies.

## Introduction

West Nile Virus (WNV), a flavivirus that was first isolated in Uganda in 1937 [1], is one of the most recent emerging mosquito-borne diseases in Europe and North America. It is maintained in a bird-mosquito transmission cycle primarily involving *Culex* species mosquitoes of which the *Cx*. *pipiens* complex is thought to be the most important in Europe [2]. Only lineage 1 and 2 of WNV have been associated with significant outbreaks in humans and horses, which act as incidental hosts in the natural transmission cycle [3]. In recent years, WNV has circulated in many European countries, including Italy, causing hundreds of human cases [4]. While most human infections are asymptomatic, about 25% of the infections develop symptoms such as fever and headache [5] and less than 1% more severe neurological diseases [6].

The first human infection due to West Nile virus lineage 2 (WNV-2) was reported in Central Italy in 2011 [7] and subsequently WNV-2 became the only strain isolated in humans and mosquitoes in the country [8]. Recent phylogenetic investigations show that WNV-2 likely entered Italy spreading from eastern European countries in which was previously introduced [9] and has settled in the Emilia-Romagna region (northern Italy) at least since 2013, causing sporadic human infections until 2017 (between 7 and 20 recorded cases per year) [10, 11]. A modelling study suggested that the most likely reactivation mechanism of the WNV transmission cycle in northern Italy is via mosquitoes which had acquired the infection the previous year, survived winter and, by taking a blood meal to begin egg production upon exiting diapausing in spring, infect susceptible birds [12]. However, as persistence in birds has been demonstrated [13], it has been suggested that in North America WNV might continue to circulate during winter in American crow populations, which can transmit the virus with fecal-oral transfer [14].

In 2018, the virus circulated earlier and more abundantly than in previous years. In fact, in the Emilia-Romagna region, WNV was detected for the first time in June, at least two weeks before the observed circulation in 2013–2017, and in a larger number of mosquito pools. Transmission resulted in 100 neuroinvasive human cases (WNND), more than the total number of cases recorded between 2013 and 2017 [15]. Here, we provide a quantitative comparison between the 2018 transmission season and previous years (2013–2017), using a transmission dynamics temperature-dependent model [12] informed by data on *Cx*. *pipiens* abundance, the main WNV vector in the area [16]. Several mathematical models for WNV transmission dynamics have been proposed, from simpler models in a constant environment [17, 18] to models that consider the dependence of parameters on environmental variables [19, 20]; in our case, the model is fitted to WNV prevalence of mosquito pools collected from the trap sites across the region during the 6 years under study, while human cases are used for validation. We eventually conjectured that the exceptionally higher 2018 spring temperatures could have helped at amplifying WNV transmission at the beginning of the season, increasing the prevalence in vector and host populations during the summer, resulting in a higher risk for human transmission.

## Methods

### Study area and entomological data

**Fig 1:**
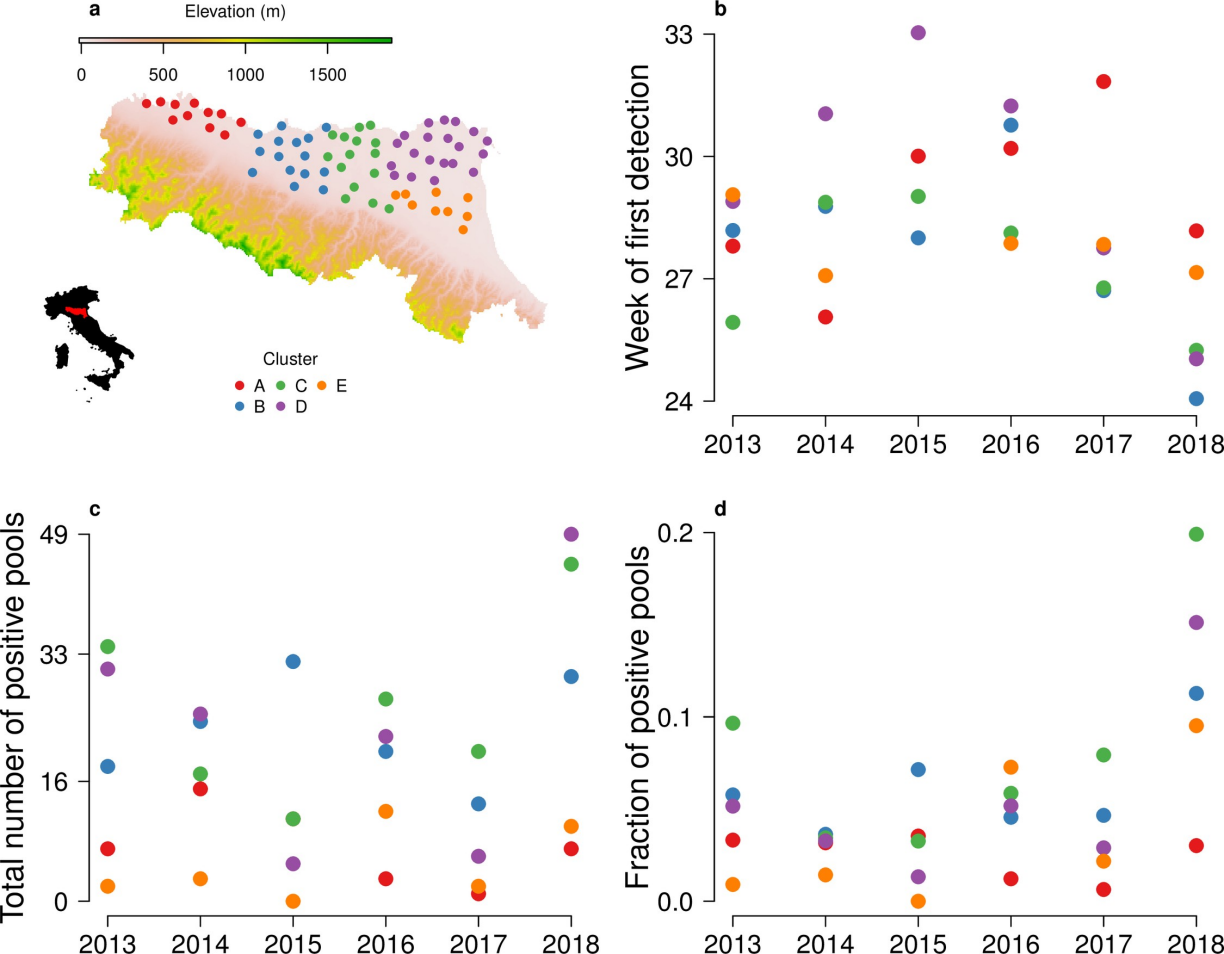
Study area and preliminary analysis. a) Study area and trap sites; b) week of first detection of a West Nile virus (WNV) positive mosquito pool in a given year; c) total number of recorded WNV positive mosquito pools; d) total fraction of recorded WNV positive mosquito pools. Colors represent the cluster division (A: red, B: blue, C: green, D: purple, E: orange). Map was generated using publicly available shapefiles [22].

Emilia-Romagna region is located in the northern part of Italy (Fig 1) and has a population of 4.46 million inhabitants. The area under surveillance is about 11,000 km^2^ wide and is located in the Po valley plain, where more than 90% of the region’s residents live, and where ecological conditions (such as *Cx*. *pipiens* breeding sites density and distribution, bird species population and environmental parameters) are considered suitable for WNV circulation [21].

The mosquito surveillance program in the study region is based on weekly captures performed between June and October each year with carbon dioxide baited traps [23] located across the area. Between 88 and 104 locations were chosen each year, to evenly monitor the entire surveillance area. Each trap site was geo-referenced and worked fortnightly for one night, from 17:00 to 9:00 the next day. Sampled mosquitoes were identified at the species level with morphological keys [24], then pooled, with a maximum of 200 specimens per pool, and screened for WNV detection via PCR [16, 21].

### Computational framework

To characterize WNV transmission in the different parts of Emilia-Romagna, we divided the region into 5 areas by clustering the trap locations (namely A, B, C, D, E in Fig 1) following a qualitative analysis of WNV circulation during the 2013–2018 transmission seasons (see section Clustering in the S1 Appendix). We then averaged the observed weekly mosquito captures among traps within the same cluster. For our analysis, in order to avoid any possible bias due to trap selection, we considered a subset consisting of 70 traps used each year between 2013 and 2018 (see Fig 1A, A: red, B: blue, C: green, D: purple, E: orange).

**Fig 2:**
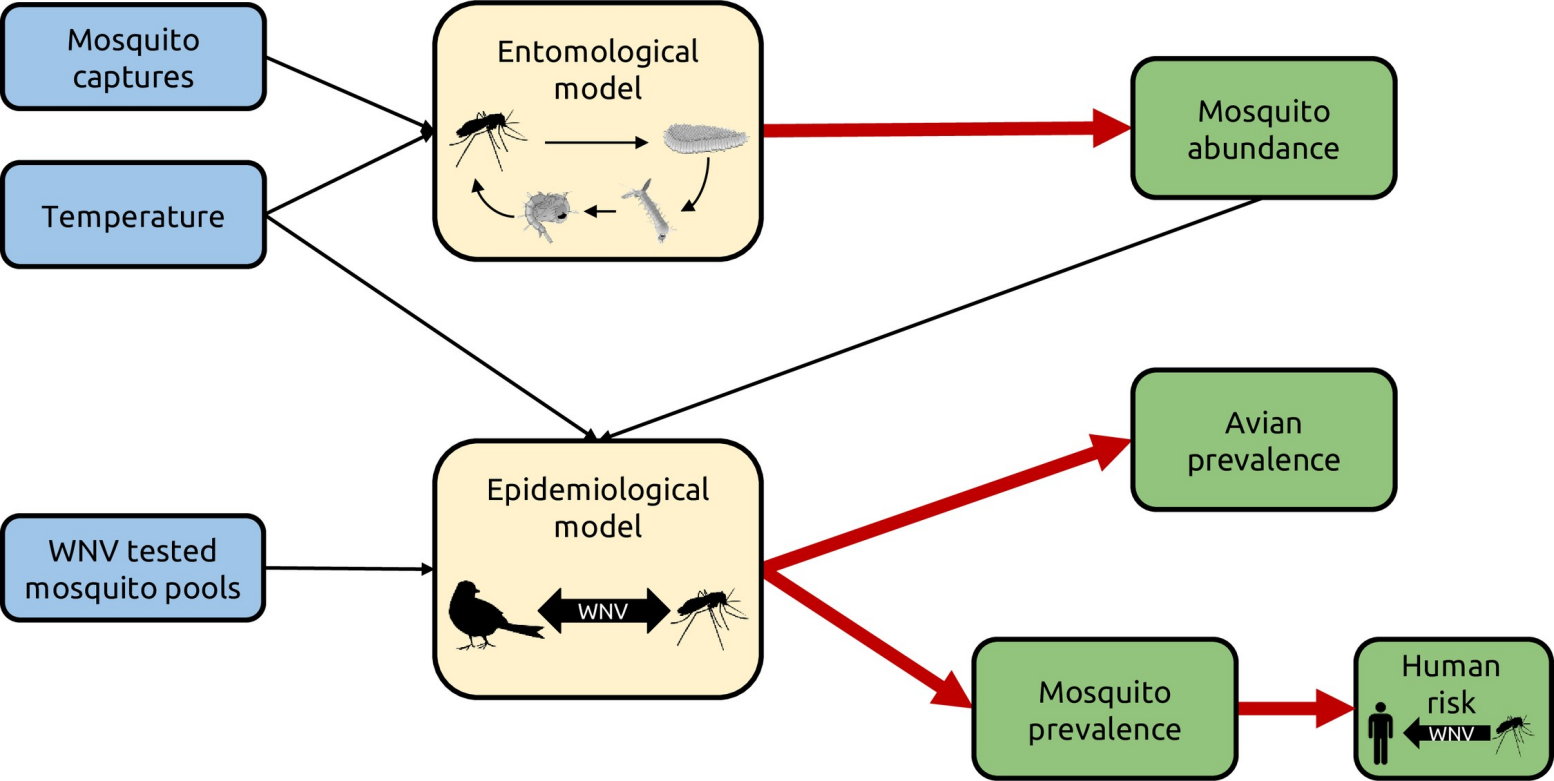
Schematic representation of the computational framework. Mathematical models (yellow boxes) take as inputs recorded temperature and entomological data (blue boxes) to estimate mosquito abundance, West Nile virus prevalences in the bird and vector populations and human transmission risk (green boxes).

Fig 2 shows a schematic representation of the computational framework adopted in this analysis. First, the averaged recorded captures were used to calibrate a mosquito population model (“entomological model”) and provide an estimate of the mosquito abundance A(*t*, *c*, *y*) for each day *t* in cluster *c* and year *y* by simulating the four life stages of *Cx*. *pipiens*, namely eggs (E), larvae (L), pupae (P) and non-diapausing female adults (A), taking explicitly into account the average daily temperature. The model is based on the following system of equations:

$$E^{\prime }=\frac{n_{E}}{d_{A}}A-(\mu_{E}+\tau_{E})E$$

$$L^{\prime }=\tau_{E}E-\left(\tau_{L}+\mu_{L}(1+\frac{L}{K_{i}})\right)L$$

$$P^{\prime }=\tau_{L}L-(\mu_{P}+\tau_{P})P$$

$$A\prime =\frac{1}{2}\tau_{P}(1-d_{P})P-\left(\mu_{A}+\chi \frac{\beta }{d_{A}}\right)A$$

where τ_E_, τ_L_, τ_P_ are the temperature dependent developmental rates driving the transitions of vectors across the different life stages considered; μ_E_, μ_L_, μ_P_, μ_A_, are the temperature dependent death rates associated with the different stages; n_E_ is the number of eggs laid in one oviposition; d_A_is the temperature-dependent length of the gonotrophic cycle; K_i_ (i = 1, 2) is the density-dependent scaling factor driving the carrying capacity for the larval stages before (i = 1) and after (i = 2) June 30; d_P_ is the probability (depending on daylight duration) that a fully developed pupa becomes a diapausing adult; β is the capture rate; *χ* is a function of the time defined equal to 1 when the trap is open and 0 otherwise. Since traps capture host seeking mosquitoes, only a fraction A/d_A_ of adults can be trapped. As only female adult mosquitoes are explicitly considered in the model, the term 1/2 in the equation for the adults accounts for the sex ratio. We allowed for two different values within each season of the density-dependent factor to take into account that during summer *Cx*. *pipiens* breeding sites availability might change, causing a possible increase in *Cx*. *pipiens* larval mortality, for instance because of competition for resources with *Ae*. *albopictus* at the larval stage [25]. The average adult mosquito densities (number of adult females per hectare) were obtained as A/*a*, where *a* is the average trapped area for every considered cluster, with *a* = π⋅*r*^2^, where *r* is the average *Cx*. *pipiens* flight range. Details on model parameters can be found in Table 1.

**Table 1:** Parameters for the entomological model.

Parameter	Interpretation	Value	Source
τ_E_	Developmental rate for eggs (day^-1^)	2.2⋅10^−3^⋅T^1.77^	[26]
τ_L_	Developmental rate for larvae (day^-1^)	$\frac{1}{93.24-6.83\cdot T+0.13\cdot T^{2}}$	[27, 28]
τ_P_	Developmental rate for pupae (day^-1^)	$\frac{1}{20.17\cdot \exp\left(-0.096\cdot T\right)}$	[27, 28]
d_A_	Gonotrophic cycle length	$\frac{1}{0.122\cdot (\log_{10}(T-9{))}^{1.76}}$	[26]
μ_E_	Death rate for eggs (day^-1^)	$0.095\cdot \exp{\left(\frac{T-22.88}{7}\right)}^{2}$	[26]
μ_L_	Death rate for larvae (day^-1^)	2.7⋅10^−2^+3⋅10^−9^⋅exp(0.64⋅T)	[27, 28]
μ_P_	Death rate for pupae (day^-1^)	2⋅10^−5^⋅(T−20.8)^4^+0.08	[27]
μ_A_	Death rate for adults (day^-1^)	$\frac{4.61}{151.6-4.57\cdot T}$	[27]
d_P_	Fraction of emerging diapausing adults	$\frac{1+\exp\left(\frac{829.86-\iota }{30.07}\right)}{1+0.5\cdot \exp\left(\frac{829.86-\iota }{30.07}\right)}-1$	[27]
n_E_	Number of eggs laid in one oviposition	200	[26, 27]
β	Daily capture rate	0.108	[29]
*r*	Daily average mosquito flight range (meters)	500	[30, 31]

T is the average daily temperature (°C), *ι* is the number of minutes of light per day.

Free model parameters to be estimated are the density-dependent factors K_1_ and K_2_ and the number of initial adults A_0_. The seasonal dynamics of the mosquito population is simulated for each cluster and year from April 1 (corresponding to approximately two months before the first capture session) to October 31. The posterior distributions of the free parameters were explored by Markov chain Monte Carlo (MCMC) sampling applied to the likelihood of observing the weekly number of trapped adults as in [27] (the likelihood formula is presented in the S1 Appendix).

We subsequently used A(*t*, *c*, *y*), the estimated average daily mosquito abundance, to reproduce the observed patterns of WNV circulation, by fitting the recorded number of WNV positive mosquito pools in year *y* and cluster *c* using a previously developed epidemiological model of WNV transmission in mosquitoes and birds [12], accounting for avian demography. At the beginning of the season the bird community is assumed to consist of adult individuals only, which can breed and reproduce until mid-July, giving birth to juvenile susceptible individuals. Susceptible birds contract the virus from bites of infectious mosquitoes. After an intrinsic incubation period, they become infectious and subsequently recover and become immune to reinfections. Susceptible mosquitoes can become exposed to infection after biting infectious birds with a temperature-dependent probability (see Table 2); in such a case, they will become infectious to the avian population after a temperature-dependent extrinsic incubation period and for the rest of their life. While originally developed for House sparrows, we adapted the demographic and epidemiological avian parameters for magpies (*Pica pica*, see Table 2), a competent host species for the virus [32] which is considered to be an important reservoir for WNV transmission in the study area [21]. A scheme of the model is presented in the S1 Appendix. The model has five free parameters: i) the initial number of adult birds (*B*_*0*_); ii) the initial fraction of immune birds (*p*_*R*_), which depends on the avian seroprevalence (i.e. the fraction of immune birds) at the end of the previous year; iii) the initial mosquito prevalence (*p*); iv-v) the vector feeding preference on birds during the first (*f*_*1*_) and second (*f*_*2*_) part of the season, to reflect *Cx*. *pipiens* preference for feeding more frequently on mammals during the second part of the season (*f*_*1*_>*f*_*2*_) [33, 34]. Each cluster *c*∈{A,B,C,D,E} and year *y*∈[2013, 2018] is simulated from May 1 to October 31 starting with B_0_(*c*, *y*) adult birds, of which a fraction p_R_(*c*, *y*) is already immune to WNV, and a fraction p(*c*, *y*) of infected mosquitoes. We considered B_0_ and p_R_ to be dependent on the previous epidemiological season. In particular, we assumed B_0_(*c*,*y*)∈[0.8⋅B_0_(*c*,*y*−1),1,2⋅B_0_(*c*,*y*−1)] and p_R_(*c*,*y*)∈[0.8⋅S_f_(*c*,*y*−1),1,2⋅S_f_(*c*,*y*−1)], where S_f_ is the average final avian seroprevalence, i.e. the fraction of recovered and immune birds at the end of the simulated season. In other words, the initial number of birds and the initial avian immunity cannot be more or less than 20% of the initial avian density and final seroprevalence of previous year respectively. Moreover, we assumed p(*c*,2013)∈[0,0.2] for *c*∈{A,B,C,D,E} since 2013 was the first year in which WNV-2 was detected in the area. The posterior distributions of the estimated parameters were explored by MCMC sampling applied to the binomial likelihood of observing the recorded number of positive pool, given the model-predicted mosquito prevalence and the actual average pool size. The complete formulas for the likelihood computation are presented in the S1 Appendix.

**Table 2:** Avian parameters for the epidemiological model.

Parameter	Value	Source
Avian fertility rate (day^-1^)	0.02 until July 15 0 afterwards	[35]
Adult birds death rate (day^-1^)	0.001	[35]
Juvenile birds death rate (day^-1^)	0.003	[35]
Length of viremia in birds (days)	5	[36]
Probability of WNV transmission from mosquito to bird per infectious bite	0.94	[37]
Probability of WNV transmission from bird to mosquito per infectious bite	$\frac{\exp\left(-10.197+0.365\cdot T\right)}{1+\exp\left(-10.197+0.365\cdot T\right)}$	[12]
Extrinsic incubation period (days)	$\frac{1}{0.0092\cdot T-0.132}$	[38]
Intrinsic incubation period (days)	2	[37]

T is the average daily temperature (°C).

Finally, we computed the human transmission risk λ_H_(*c*, *w*, *y*) as the probability, for a person living in a given cluster *c*, of being bitten by an infectious mosquito in a given week *w* of year *y* as in [12]. To summarize, λ_H_ is the ratio between the number of bites given to not competent hosts (1-*f*_*i*_) by infectious mosquitoes (whose abundance was estimated previously with the epidemiological model) and the number of humans living in the area surrounding the trap. We then used λ_H_ to predict the number of reported WNV human infections for each cluster and year by fitting the observed number of cases from a Poisson(H_c_∙ρ_c_∙ λ_H_(*c*, *w*, *y*)), where H_c_ is the number of human beings living in cluster *c* and ρ_c_ is a cluster-dependent free rescaling parameter, estimated with a MCMC approach applied to the Poisson likelihood of observing the recorded infections, given the model predictions, by modelling together the six considered years. All details can be found in the Human transmission section of the S1 Appendix.

## Results

In 2018, WNV was recorded earlier (June) and in a higher fraction of pools in the two central (B, C) and two northeastern (D, E) clusters with respect to previous years. More specifically, between 2013 and 2017 WNV was first detected in mosquito pools between the 26^th^ and 33^th^ week of the year, according to the cluster; on the other hand, in 2018 the first WNV positive pools were recorded between the 24^th^ and 25^th^ week in three out of the five clusters (B, C and D).

**Fig 3:**
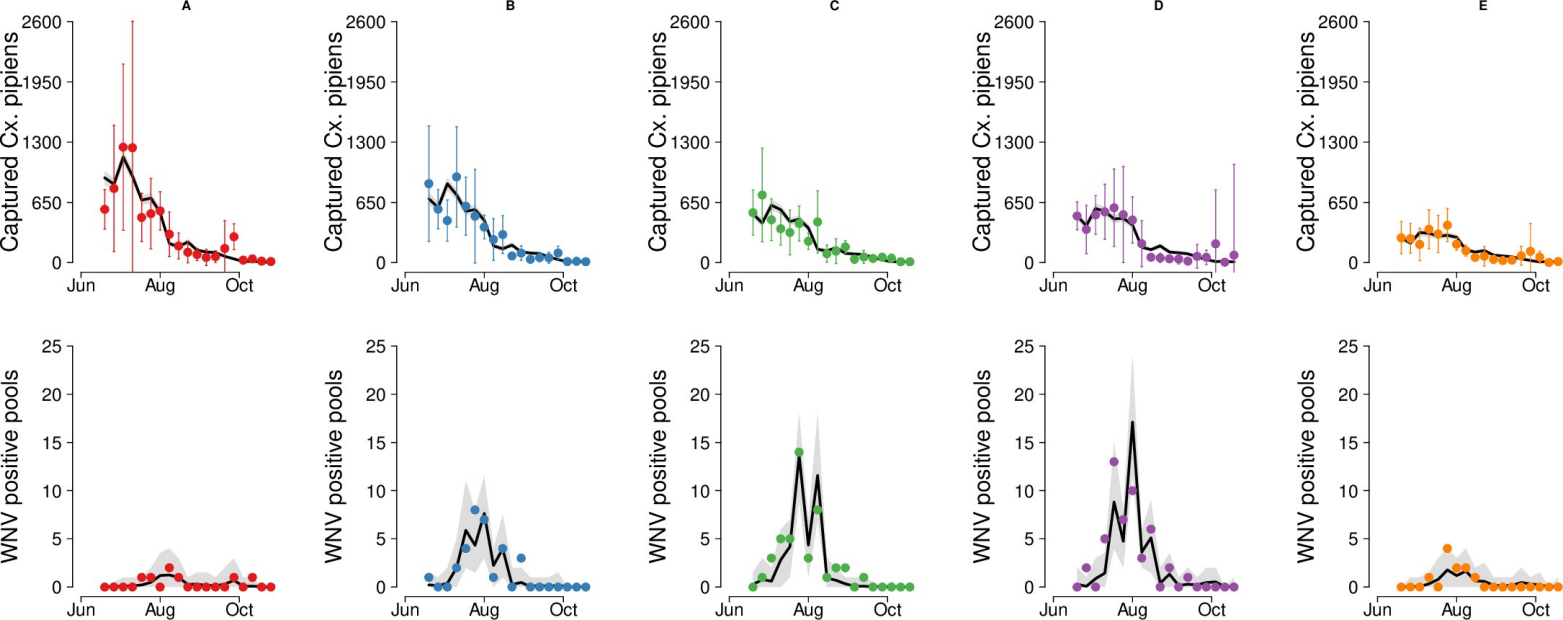
Models fit for 2018. Fit of the entomological (first row) and epidemiological (second row) models to the observed average number of captured *Cx*. *pipiens* mosquitoes and total number of WNV positive pools in 2018 by cluster (A: first column, B: second column, C: third column, D: fourth column, E: last column). Solid line: average. Shaded area: 95%CI of model predictions. Dots: observed data. Bars: 95%CI for the average of the observed captures.

The mosquito captures and pool fits for 2018 are shown in Fig 3. Considering all years under study, we found that 86% of the 95% confidence intervals (CI) of the averaged recorded entomological overlaps the 95% CI predictions of the entomological model, while 96% of the total number of weekly positive pools lie within the 95% CI predictions of the epidemiological model.

Note that the estimated parameters suggest that indeed a change in mosquito feeding preferences and demography systematically occurred every season; in fact, during the second half of the breeding season *Cx*. *pipiens* larval survival is estimated to decrease (see Figure C in the S1 Appendix), while female adults feed less on avian hosts (see Figure E in the S1 Appendix).

**Fig 4:**
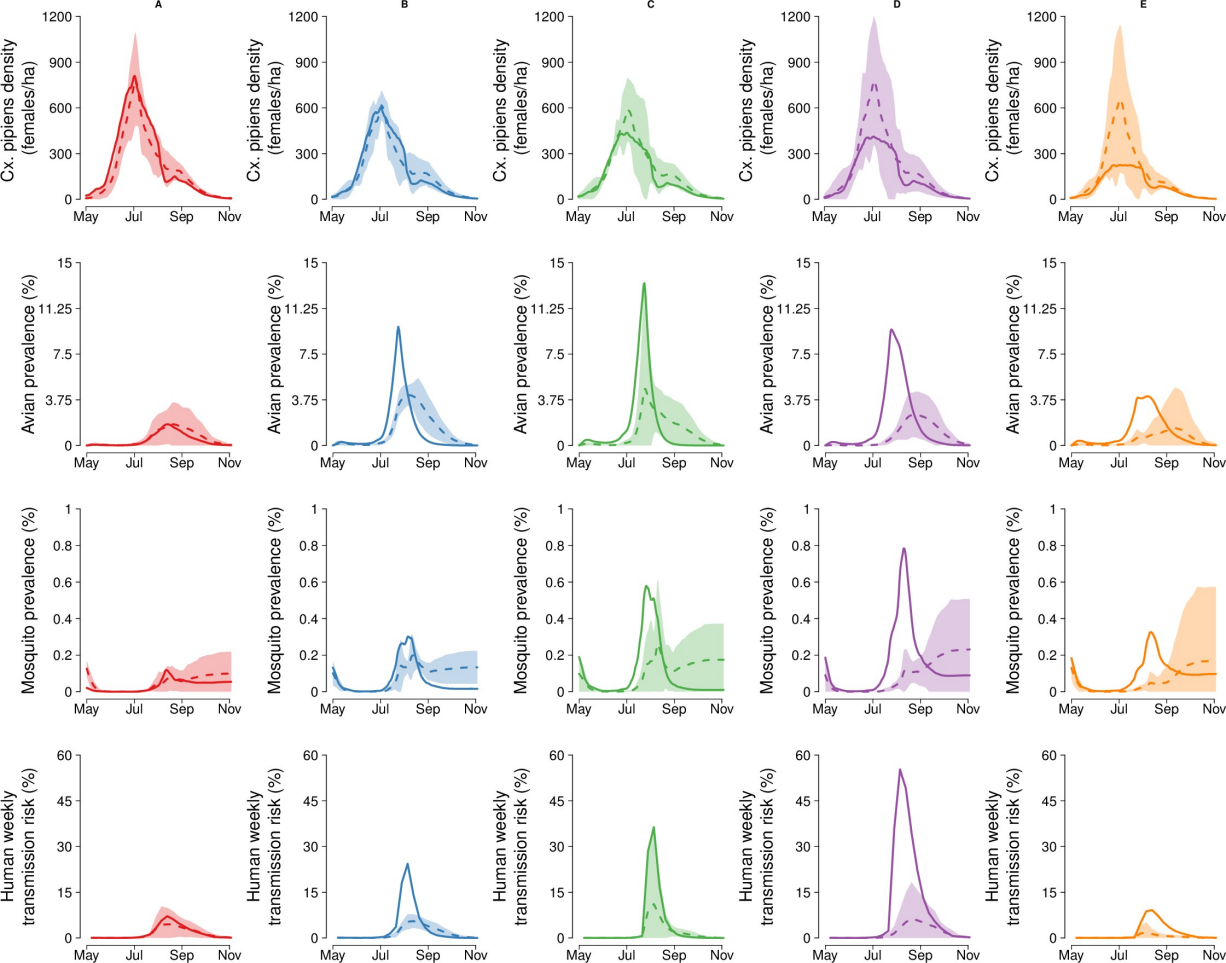
Comparison between 2018 and previous seasons. Comparison between 2018 (continuous lines) and averaged previous seasons (2013–2017, dashed lines, shaded areas represent the 95%CI of the averaged 2013–2017 seasons) of the mean i) estimated mosquito abundance (number of adult females per hectare, first row), ii) avian prevalence (second row), iii) mosquito prevalence (third row) and iv) λ_H_, the human transmission risk, (bottom row) for each cluster (A: first column, B: second column, C: third column, D: fourth column, E: last column).

The estimated average mosquito density in 2018 was either similar (clusters A-C) or lower (clusters D-E) than the 2013–2017 average (Fig 4, first row). By fitting the observed number of positive mosquito pools, the epidemiological model reconstructs the daily *Cx*. *pipiens* WNV prevalence and provides an estimate of the infection rate in the avian population. The prevalence of WNV was much higher in 2018 with respect to 2013–2017 seasons, especially in the eastern regions, (Fig 4, second and third row), reaching a maximum value of about 15% for the bird population in cluster C and 0.8% for the mosquito population in cluster D towards the end of July. In this latter area, estimated maximum mosquito and avian prevalences for 2018 were more than three times the maximum of the 2013–2017 average. WNV transmission was also temporally different in 2018 in the host population; indeed, in some clusters the peak avian prevalence occurred earlier, corresponding to a steeper decline towards the end of summer resulting in a lower average final infection (clusters B, C and D) during the autumnal months. Such final lower prevalence was predicted also in the mosquito population, especially in clusters B and C.

Moreover, for regions C and D the final bird seroprevalence for 2018 was estimated to be quite higher than in previous years; in fact, while the majority of the predicted averages ranged between 32% and 69%, according to the cluster and the years 2013 to 2017, in 2018 estimated averages were about 80% (see Figure F in the S1 Appendix).

We found a much higher predicted risk for human transmission in 2018, especially for cluster D, where its peak was evaluated to be more than eight times higher than previous seasons (Fig 4, last row). The model estimates a higher WNND occurrence in 2018 and, overall, 80% of the number of cases recorded for each cluster and year lie within the 95% CI predictions (see section Human transmission in the S1 Appendix).

**Fig 5:**
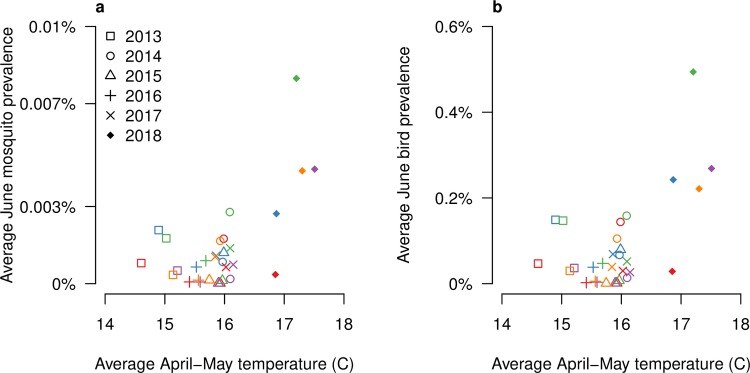
Estimated prevalences VS spring temperatures. Estimated average June mosquito (panel a) and avian (panel b) prevalence (Y-axis) versus spring average temperatures in degrees Celsius (X-axis). Colors represent the cluster division (A: red, B: blue, C: green, D: purple, E: orange).

Finally, we found a positive statistically significant (p-values<0.001) correlation (Pearson’s correlation coefficient 0.62 and 0.58 respectively) between avian and mosquito average prevalence in June and the average April-May temperature (Fig 5). In particular, we noted that estimated prevalences and recorded temperatures in 2018 were significantly higher (Student’s t-test p-value<0.001) than in previous years.

## Discussion

In this study, we analyzed the dynamics of WNV in the Emilia-Romagna region, northern Italy, using previously developed mathematical models to estimate mosquito density and simulate virus transmission between mosquitoes and birds. Both models replicated closely the observed patterns of dynamics and infection and allowed us to quantitatively assess the differences in avian and mosquito prevalence and human transmission risk between 2018 and the previous seasons (2013–2017). We found that the estimated prevalence in 2018 was much higher than in previous years, especially in the eastern part of the study region. The risk for a human of being bitten by an infectious mosquito was evaluated to be substantially greater during 2018, and this finding is consistent with the larger number of recorded WNND cases.

Which could be the factors responsible for the large increase in WNV circulation observed in 2018? In principle, it might depend, for instance, on a higher abundance of mosquitoes, or on a higher susceptibility in avian populations, or on mutations in viral properties that allowed for a higher probability of transmission, or on environmental changes in 2018.

However, mosquito densities were comparable or lower than in previous years. Furthermore, higher mosquito densities are not straightforwardly associated to a greater WNV circulation. For instance, in Romania, a study carried out over 3 consecutive years did not demonstrate a significant association between mosquito density and infection rates [39]. Similarly, in the US, vector abundance was not found to be a good predictor for human infection [40, 41]. Finally, a study carried out over a larger area partially encompassing Emilia-Romagna did not detect a strong relationship between mosquito abundance and viral circulation [16].

We did not find any remarkable difference in the distribution of the estimated free parameters of the epidemiological model between 2018 and the previous years, although we can note that the initial mosquito prevalence was estimated slightly higher for 2018 for some clusters. The estimated avian seroprevalence at the end of 2017 was comparable to the previous years, and such comparison holds for summer temperatures as well (see Figures B, E and F in the S1 Appendix).

On the other hand, spring was exceptionally warm in 2018 with respect to previous years: while between 2013 and 2017 the average April-May temperature varied between 14.6 and 16.1°C across the different clusters, it ranged between 16.9 and 17.5°C in 2018. We found a significant positive relationship between the estimated avian and mosquito prevalence and spring temperatures. It is well known that temperature plays a key role in the WNV transmission cycle, as it affects several epidemiological parameters such as the extrinsic incubation period [38], the biting rate [26] and especially the transmission probability [42]: an increase of two degrees (from 15.5°C to 17.5°C) doubles the host-to-vector transmission probability (from 0.005 to 0.01 [12]).

It appears then likely that spring temperature, the only substantially different environmental factor between 2018 and the previous years, has played a key role in amplifying WNV transmission at the beginning of the season. Similar conclusions were found in [39, 43–45], where statistical analyses suggested that early spring temperature conditions may be particularly important for activating the mosquito breeding season, reducing the extrinsic incubation period and thus accelerating virus amplification in the avian and mosquito populations. However, other factors could play an important role as well and should therefore not a priori ruled out. For instance, a high avian immunity at the beginning of the epidemiological season, due to the previous year WNV circulation, might prevent pathogen transmission. Other climatic variables not explicitly considered in our models, such as drought, precipitation or winter temperature might influence WNV transmission as well [39, 44–49]. Nonetheless, it is interesting to note that average April-May temperatures in the region were rather low in 2019 (see S1 Appendix), and WNV human transmission in the same year has been very limited; in fact, only 4 WNND infections have been reported in Emilia Romagna so far, all in the province of Modena [50].

Since an important feature of the model used is the function relating host-to-vector transmission probability to temperatures, we conducted a sensitivity analysis to assess how uncertainties in the specific form on the function affect the results; we thus tested perturbed functions fitting the data presented in [42], and found that the model is robust to perturbations of the relationship assumed. In fact, perturbations on this function produce small variations in the model simulations (see section Sensitivity analysis in the S1 Appendix). Moreover, we conducted the analyses by including all visited traps, obtaining very similar results (not reported).

The model used has definitely several limitations, one of the most relevant being the use of a single compartment of birds as competent hosts, although several competent bird species are present in the region [37, 51, 52]. Some mathematical models have included more than one host species, with differential preference by mosquitoes [53–55]; however, it is difficult to apply them in the specific situation, as it is unclear which bird species are most relevant for WNV transmission in Europe [56, 57]. We investigated whether assuming a different avian population (in particular, consisting of house sparrows instead of magpies) would significantly affect our findings (see section Sensitivity analysis in the S1 Appendix). Such assumption resulted in a worse fit of the recorded positive pools and in a lower estimate of both mosquito and avian prevalence, consistently with the shorter viremia of this species, which lasts about two days instead of five. Nonetheless, we still found a positive significant correlation between spring temperature and June mosquito prevalence, confirming the important role of this factor at shaping WNV transmission.

We remark that our estimates for the human transmission risk do not represent absolute values. They are computed assuming, for the sake of simplicity, that a fraction *f*_*i*_ of the mosquito bites are distributed on competent avian hosts, and the complementary (1-*f*_*i*_) on humans. Realistically, there could be many other non-competent hosts, either birds or mammals, on which *Cx*. *pipiens* might feed. Thus, such estimated risks should be interpreted as relative measures, as they provide a metric allowing to compare quantitatively different clusters and years. The overall correspondence (Figures M and N in the S1 Appendix) between predicted risk and observed human cases suggests that the model predictions can provide useful tools for public health. Observing the figures, one may note that the number of cases observed in 2018 in cluster C appears significantly larger than predicted. Although it is expected in a statistical model that some observations may differ greatly from predictions, we thought it worth looking at data in greater detail, and found out that more than one third of the 2018 human cases of cluster C occurred in the city of Bologna, the largest center in the region. This suggests that perhaps the model parameters, which were fitted to average conditions of the region, should be modified in large urbanized areas, where ecological settings might differ significantly with respect to more rural zones.

Despite the limitations, this study provides new important insights into the ecology of WNV in southern Europe and represents a first quantitative assessment of the dependency between temperature and infection that can explain why WNV circulation in 2018 was much higher than in previous years. Spring temperature is among the crucial factors for WNV amplification, and temperatures like in 2018 are likely to become more common under the projected scenarios of climate change [58]. Thus, weather anomalies at the beginning of the mosquito breeding season might act as an early warning signal for public health authorities, enabling them to design efficient surveillance and prevention strategies.

## Supporting information

S1 Appendix Supporting text containing methodological details and additional results. (PDF)

S1 Table Recorded average entomological captures for each year and cluster. (XLSX)

S2 Table Total number of analyzed mosquito pools for each year and cluster. (XLSX)

S3 Table Total number of WNV positive pools for each year and cluster. (XLSX)
