# Supplementary material for: A quantitative comparison of West Nile virus incidence from 2013 to 2018 in Emilia-Romagna, Italy
Source: PLoS Negl Trop Dis. 2020 Jan 2;14(1):e0007953. doi: 10.1371/journal.pntd.0007953 (PMC6939904; doi:10.1371/journal.pntd.0007953)
Supplement: S1 Appendix — (PDF) [file pntd.0007953.s001.pdf]

A quantitative comparison of West Nile virus incidence from 2013 to 2018 in Emilia-Romagna, Italy

Giovanni Marini[1], Mattia Calzolari[2], Paola Angelini[3], Romeo Bellini[4], Silvia Bellini[2], Luca Bolzoni[2], Deborah Torri[2], Francesco Defilippo[2], Ilaria Dorigatti[5], Birgit Nikolay[6,7,8], Andrea Pugliese[9], Roberto Rosà[1,10,11], Marco Tamba[2]

[1]Department of Biodiversity and Molecular Ecology, Research and Innovation Centre, Fondazione Edmund Mach, San Michele all'Adige, Italy
[2]Istituto Zooprofilattico Sperimentale della Lombardia e dell'Emilia Romagna "B. Ubertini", Brescia, Italy
[3]Regione Emilia-Romagna, Bologna, Italy
[4]Centro Agricoltura Ambiente "G. Nicoli", Crevalcore, Italy
[5]MRC Centre for Global Infectious Disease Analysis, Department of Infectious Disease Epidemiology, Imperial College London, London, United Kingdom
[6]Mathematical Modelling of Infectious Diseases Unit, Institut Pasteur, Paris, France
[7]CNRS UMR2000: Génomique évolutive, modélisation et santé, Institut Pasteur, Paris, France
[8]Center of Bioinformatics, Biostatistics and Integrative Biology, Institut Pasteur, Paris, France
[9]Department of Mathematics, University of Trento, Italy
[10]Epilab-JRU, FEM-FBK Joint Research Unit, Province of Trento, Italy
[11]Center Agriculture Food Environment, University of Trento, San Michele all'Adige (TN), Italy

S1 Appendix

Table of contents

Clustering 2
Temperature trends 3
Additional results 4
Sensitivity analysis 8
Human transmission 13
References 15

Clustering

To subdivide the monitored area in uniform zones, we consider data from single sites (or very close sites) sampled all years from 2013 to 2018. The number of years with at least one WNV-positive pool was considered as indication of persistence of the virus in a particular site. By interpolating these values (multilevel b spline interpolation) we obtained the map shown in Figure A, which allows to define areas with comparable persistence during the years, and led to the cluster division proposed in the main text. Exactly four sites near the center of the trapping region have been positive every year. When looking at the weekly pattern of incidence in the four sites within each year, it was clear that the easternmost two and the westernmost two were highly correlated to each other (they were both positive or both negative most bi-weeks), while the two pairs tended to be positive in different weeks of the year. In order to detect the spread pattern occurring every year, we then kept these in separate clusters (B and C); we included in each of these clusters the sites spatially close to them, with an overall pattern of positive weeks not too different. After this choice, the remaining subdivision followed. Western-most sites have a pattern of incidences quite different from other sites and similar between them. As for the eastern sites, it is clear from Figure A that the northern ones were more often positive than the southern ones; thus, we formed two other clusters, a northeastern and a southeastern (see Fig. 1a in the main text for the map of clusters).

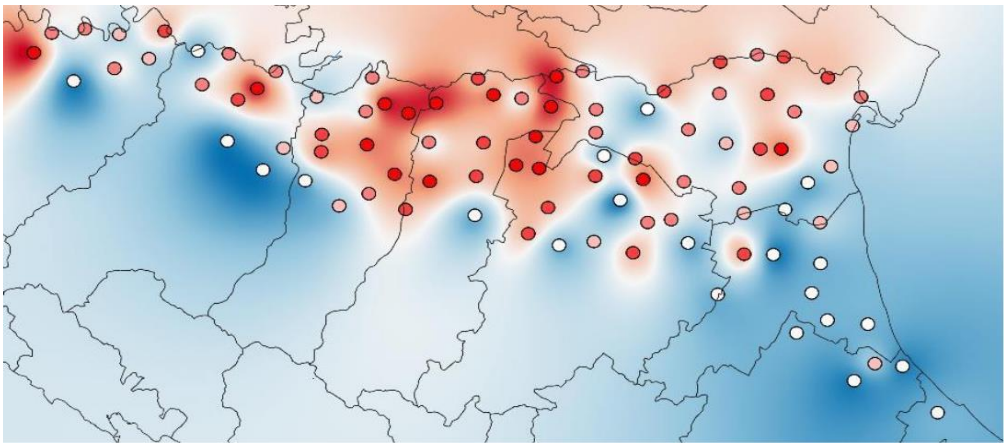

Figure A. Traps interpolation according to the number of years with positive WNV detection.

Temperature trends

The daily average temperatures were provided, for every trap site, by the Regional Environmental Agency (ARPAE Emilia-Romagna) according to a high-resolution gridded interpolated dataset [1]. Figure B compares the daily 2018 mean temperature with the daily average computed between 2013 and 2017.

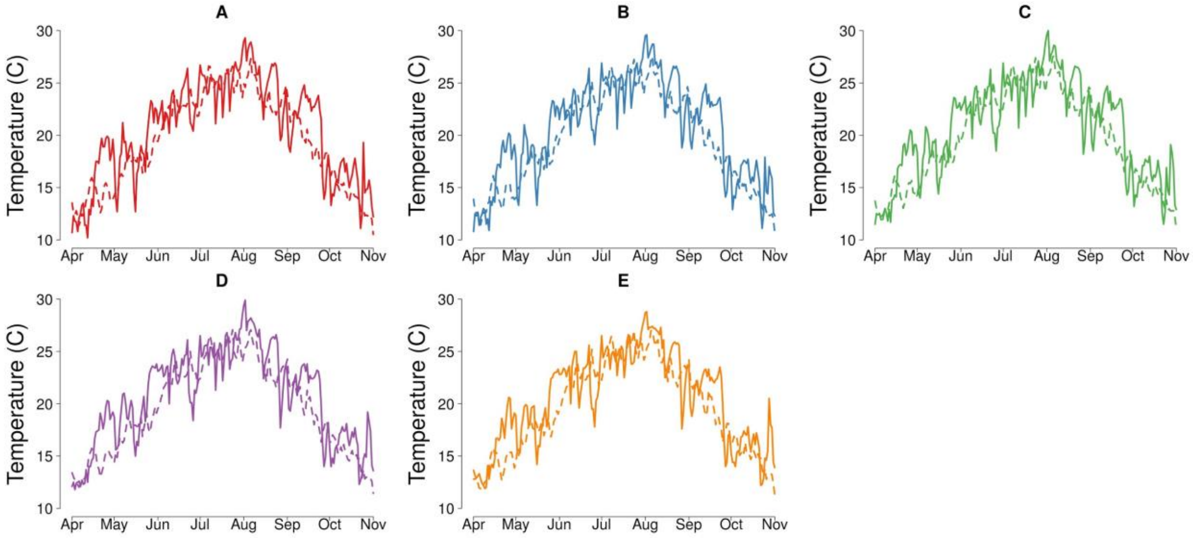

Figure B. Average daily temperature for each cluster. Continuous line: 2018 data. Dashed line: average over 2013-2017.

Average April-May temperatures for 2019, collected from [2], ranged between 14.4 and 14.7 °C in the major cities (Bologna, Ferrara, Modena, Parma, Piacenza, Ravenna, Reggio nell'Emilia and Rimini) of the study area.

Additional results

Entomological model

Assuming that the number of observed trapped adult mosquitoes follows a Poisson distribution with mean obtained from the model, similarly to [3], the likelihood of the observed data for a given cluster *c* and year *y* has been defined as

$$L_{ent}(c,y) = \prod_{s=1}^{N_c(y)} e^{-\tilde{C}(s,c,y)} \cdot \frac{\tilde{C}(s,c,y)^{n(s,c,y)}}{n(s,c,y)!}$$

where *s* runs over the considered sampling dates, $N_c(y)$ is the number of samplings for cluster *c* during year *y*, *n*(*s*, *c*, *y*) is the observed average number of trapped adult female mosquitoes over the sites of cluster *c* for sampling *s* during year *y* and $\tilde{C}(s,c,y)$ is the predicted number of captures simulated by the model with parameters $\{A_0, K_1, K_2\}$.

We allowed for two different values within each season of the density-dependent factor in the entomological model to take into account that during summer *Cx. pipiens* breeding sites availability might change, causing a possible increase in *Cx. pipiens* larval mortality, for instance because of competition with *Ae. albopictus* [4]. Indeed, as shown in Figure C, scaling factors are estimated to decrease during the second part of the breeding season, resulting in a lower larval survival.

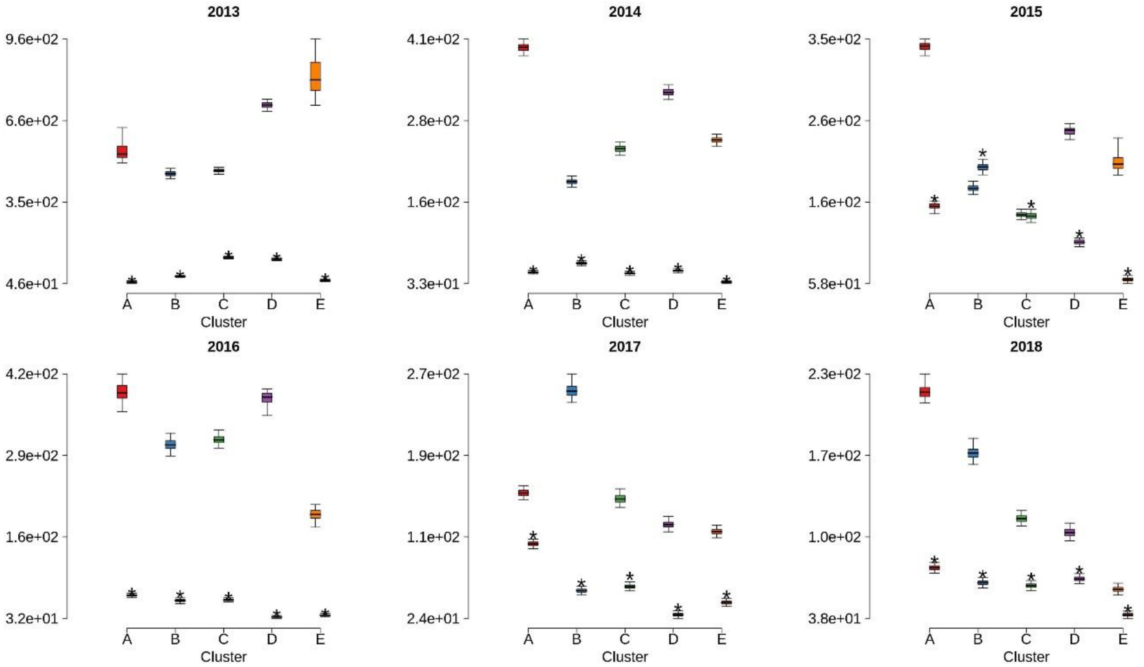

Figure C. Boxplots (2.5%, 25%, 75% and 97.5% quantiles and median) of the estimated distributions of the density-dependent scaling factors K_1 and K_2 for each cluster and year under study. Distributions for K_2 (scaling factor after June 30) are identified by an asterisk.

Epidemiological model

The model we used to simulate WNV transmission in an avian population consisting of adult and juvenile individuals can be represented with the scheme shown in Figure D.

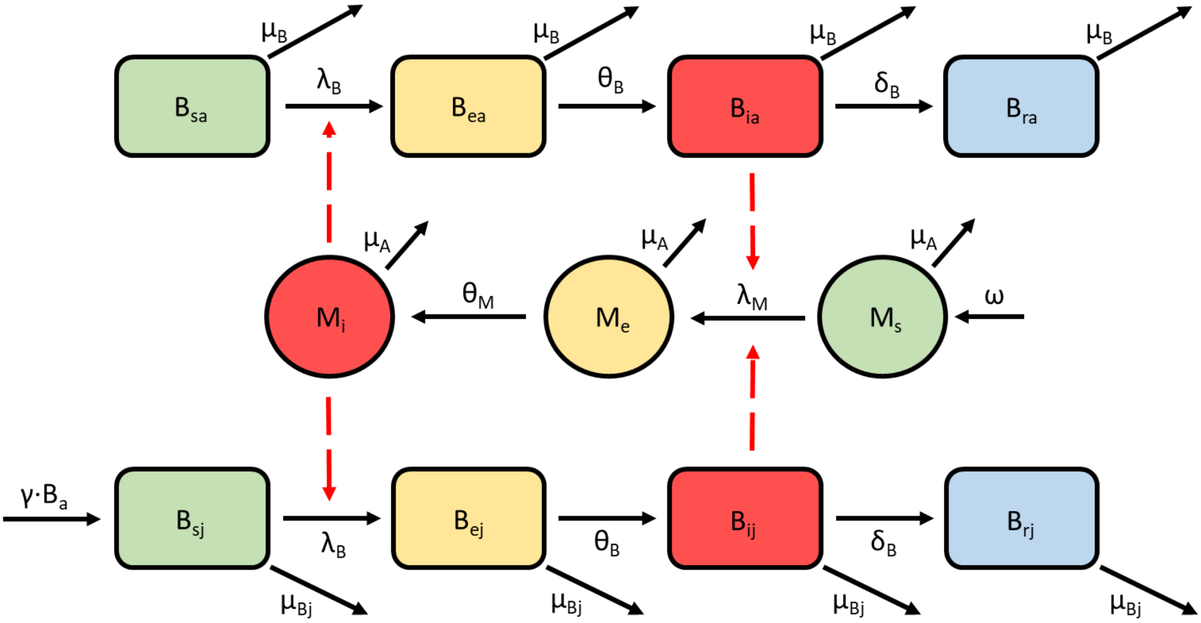

Figure D. Model flow chart for WNV transmission in birds (squares) and mosquitoes (circles) in an average trapped area. Compartments: B_{sa}, B_{ea}, B_{ia}, B_{ra} (B_{sj}, B_{ej}, B_{ij}, B_{rj}): adult (juvenile) susceptible, exposed, infectious and immune birds; M_s, M_e, M_i: susceptible, exposed and infectious mosquitoes.

At the beginning of the season the bird community is assumed to consist of adult individuals only, which can breed and reproduce until mid-July, giving birth to juvenile individuals with rate γ. Susceptible adult (juvenile) birds B_{sa}(B_{sj}) contract the virus from bites of infectious mosquitoes with overall probability λ_B (which takes into account the biting and the transmission rates). After an intrinsic incubation period θ_B, they become infectious and subsequently recover, with rate δ_B, and become immune to reinfections. Susceptible mosquitoes (M_s) can become exposed to infection (M_e) after biting infectious birds with an overall probability λ_M which takes into account the temperature-dependent biting and transmission rates; in such a case, they will become infectious to the avian population (M_i) after a temperature-dependent extrinsic incubation period $1/\theta_M$ and for the rest of their life. Here, we did not model explicitly the mosquito dynamics since we had previously estimated *Cx. pipiens* abundance through the entomological model. So, we considered the total number of adult mosquitoes, $M(t)$, as a known function; specifically, for each cluster c and year y, $M(t)=A(t, c, y)$ (see main text). If $\omega(t)=M(t)-(M_s(t)+M_e(t)+M_i(t))>0$, then at day t new $\omega(t)$ susceptible mosquitoes enter the system. A more comprehensive explanation of the model can be found in [5], while parameters values are presented in Table 2 in the main text.

The likelihood formula used for the MCMC calibration of the epidemiological model is

$$L_{epi}(c,y) = \prod_{s=1}^{N_c(y)} \binom{N_c(s,y)}{K_c(s,y)} P_c(s,y,\Psi)^{K_c(s,y)} (1 - P_c(s,y,\Psi))^{N_c(s,y)-K_c(s,y)}$$

where *s* runs over the considered sampling dates of cluster *c* during year *y*, $N_c(y)$ is the number of samplings for cluster *c* during year *y*, $K_c(s, y)$ is the number of recorded positive pools for sampling *s* for cluster *c* during year *y* and

$$P_c(s, y, \Psi) = 1 - \left(1 - \frac{M_i(s,\Psi)}{M(s,\Psi)}\right)^{u_c(s)},$$

represents the probability that at least one mosquito in the pool is infected. Here $M_i(s, \Psi)$ and $M(s, \Psi)$ are respectively the number of infected and total mosquitoes predicted by the model for the sampling date *s* (for cluster *c* during year *y*) with free parameters $\Psi=\{B_0(c, y), p_R(c, y), p(c, y), f_1(c, y), f_2(c, y)\}$ and $u_c(s)$ is the average of the pool sizes analysed on day *s*.

As shown in Figure E, the estimated distributions for the epidemiological free parameters for 2018 are compatible with the ones found for previous seasons, although we can note that for some clusters (e.g. C, D and E) the initial mosquito prevalence *p* is estimated to be slightly higher.

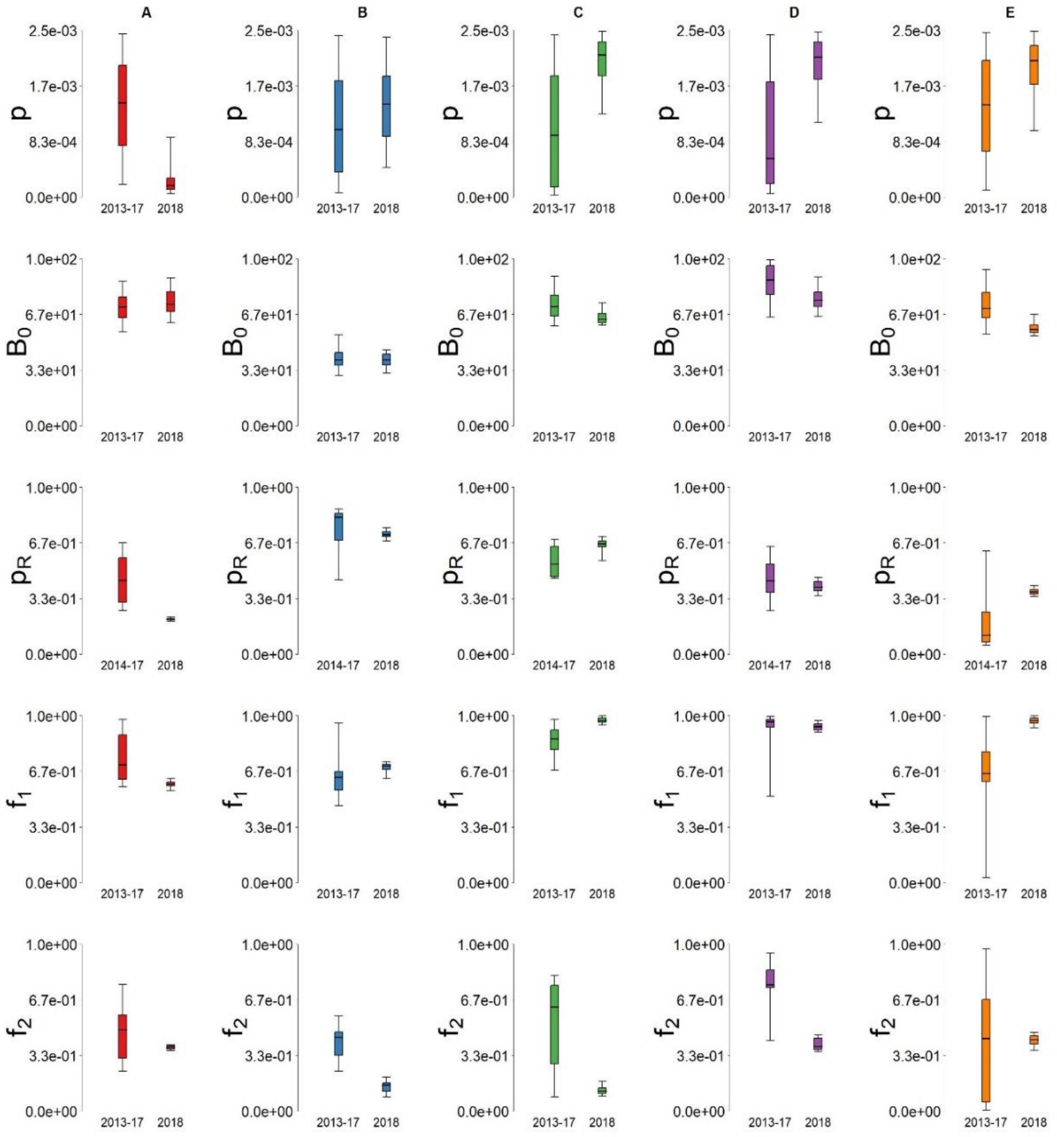

Figure E. Boxplots (2.5%, 25%, 75% and 97.5% quantiles and median) of the estimated distributions of the free parameters of the epidemiological model: p, B_0, p_R, f_1, f_2 (from first to last row) for each cluster A, B, C, D, E (from first to last column). Distributions are shown aggregating years before 2018.

Figure F shows for each cluster and year the average final avian seroprevalence S_f, i.e. the fraction of immune birds predicted at the end of the season. We can note that the S_f is quite high for 2018 for clusters with a very high WNV circulation (B-D).

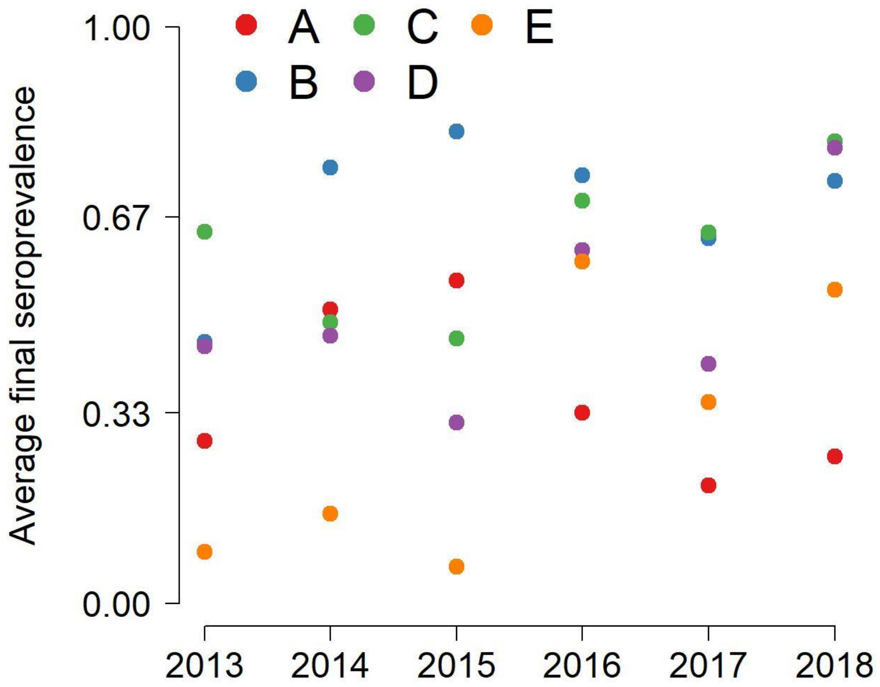

Figure F. Final average seroprevalence estimated by the epidemiological model per year and cluster.

Sensitivity analysis

Host-to-vector transmission probability

In the epidemiological model, p_{BM}, the probability that a susceptible mosquito acquires the infection from an infected bird, is a temperature dependent function [5] defined as

$$p_{BM} = \frac{\exp(a + b \cdot T)}{1 + \exp(a + b \cdot T)},$$

with *a* and *b* obtained with a binomial regression model computed on the *Cx. pipiens* competence observed upon laboratory infection at three different temperatures (namely 18, 23 and 28 °C) [6]. Because of this relatively small number of observations, we decided to perform some sensitivity analysis on the model by perturbing such function as follows: let *m*=(*a*, *b*) and *S* the covariance matrix of the estimated coefficients. Then we computed a new function with coefficients *m*'=(*a*', *b*') with

$$m' = m + A \cdot X$$

Where *X* is s standard normal distribution N(0,1) and *A* is such that $A \cdot A^t = S$. In this way,

$$var(m') = A \cdot var(X) \cdot A^t = A \cdot A^t = S.$$

We generated 100 new p_{BM} functions, which are presented in Figure G, and, for each of them, we calibrated the epidemiological model as explained previously.

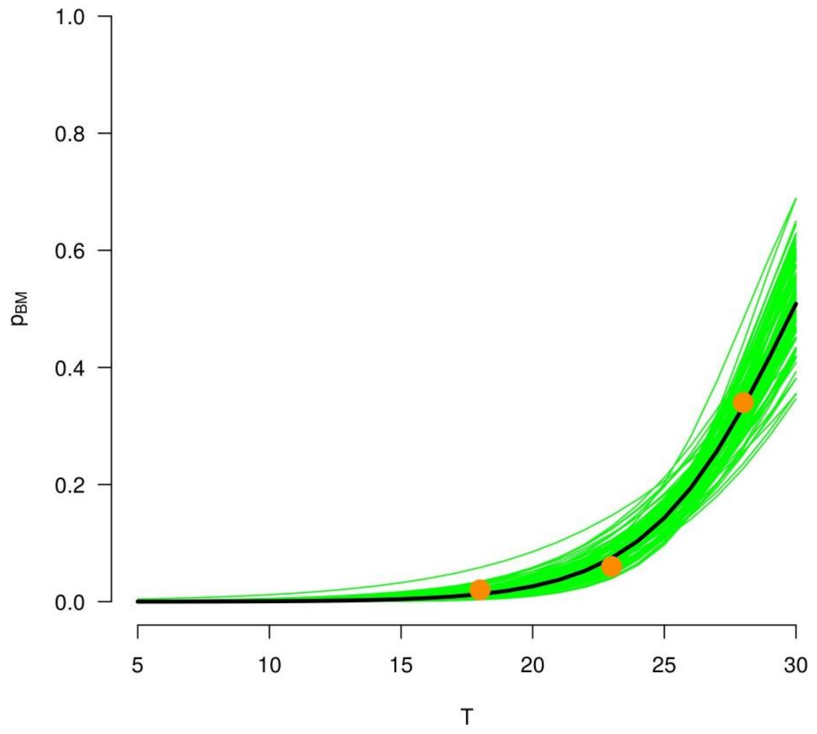

Figure G. Perturbations of the host-to-vector transmission probability function. Orange points: observed probability from laboratory experiment [6]; black lines: baseline function [5]; green lines: perturbed functions used for the sensitivity analysis.

As shown in Figure H, we did not find any remarkable difference between the estimated posterior distributions of the model free parameters. Such consistency can be observed in the model predictions as well. For instance, as shown in Figure I, estimated avian and mosquito prevalence for 2018 with the modified transmission probability are very similar to the baseline ones.

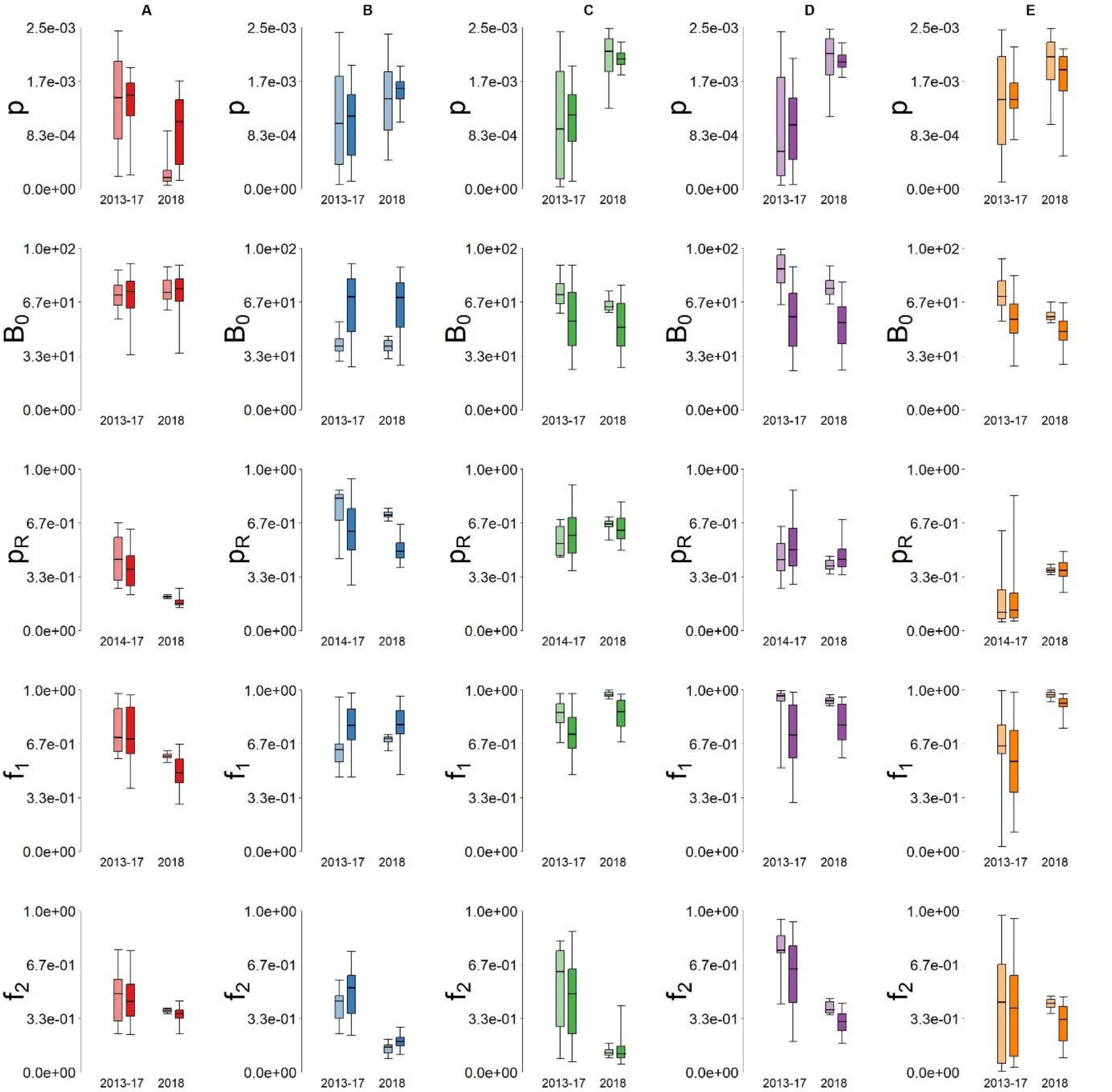

Figure H. Boxplots (2.5%, 25%, 75% and 97.5% quantiles and median) of the estimated distributions of the free parameters of the epidemiological model: p, B_0, p_R, f_1, f_2 (from first to last row) for each cluster A, B, C, D, E (from first to last column). Lighter colors show baseline distributions (see Figure C) while darker colors show estimates obtained by perturbing the bird-to-mosquito transmission probability function in the epidemiological model. Distributions are shown aggregating years before 2018.

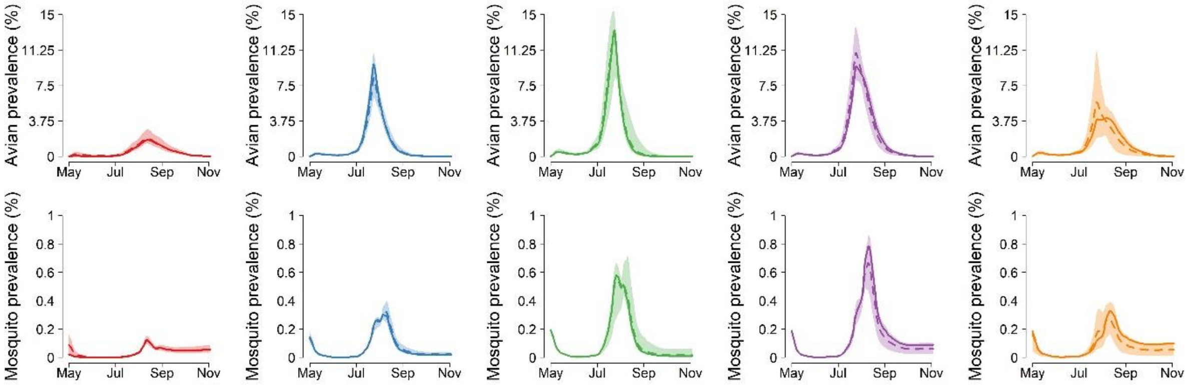

Figure I. Comparison between estimated average avian (first row) and mosquito (second row) prevalence with the baseline epidemiological model (continuous line) and by perturbing the bird-to-mosquito transmission probability function (dashed lines) for each cluster A, B, C, D, E (from first to last column). Shaded areas represent the 95%CI of simulations with the modified function.

House sparrow model

We calibrated our epidemiological model assuming the avian population consists of magpies, which are thought to be one of the main reservoirs for WNV transmission in the study area [7]. To investigate whether modelling a different bird species would affect significantly our results, we re-calibrated the model adapting as done in [5] the avian demographic and epidemiological parameters for house sparrow (*Passer domesticus*), a competent host for WNV [8]. In particular (see Table A), infectious sparrows recover faster than magpies.

Table A. House sparrow parameters for the epidemiological model.

Parameter	Value	Source
Avian fertility rate (day^{-1})	0.05 until July 15<br>0 afterwards	[9]
Adult birds death rate (day^{-1})	0.0015	[9]
Juvenile birds death rate (day^{-1})	0.0083	[9]
Length of viremia in birds (days)	1.8	[8]

This model performed slightly worse, as only 87% (compared to the 96% obtained with the baseline model) of the total number of weekly positive pools lie within the 95% CI predictions. As shown in Figure J, the posterior distributions of some of the model free parameters are quite different. For instance, the initial number of house sparrows (B_0) is estimated to be smaller with respect to magpies, while *Cx. pipiens* is predicted to feed more on this bird species, possibly because of its lower estimated abundance. The predicted prevalence is lower as well, especially in the host population, consistently with the assumed shorter viremia (see Figure K). We still found a positive statistically significant (p-value=0.04) correlation (Pearson's correlation

coefficient 0.37) between mosquito average prevalence in June and the average April-May temperature (see Figure L, panel a), while for the avian prevalence (see panel b) it is not significant (Pearson's correlation coefficient 0.34, p-value=0.06).

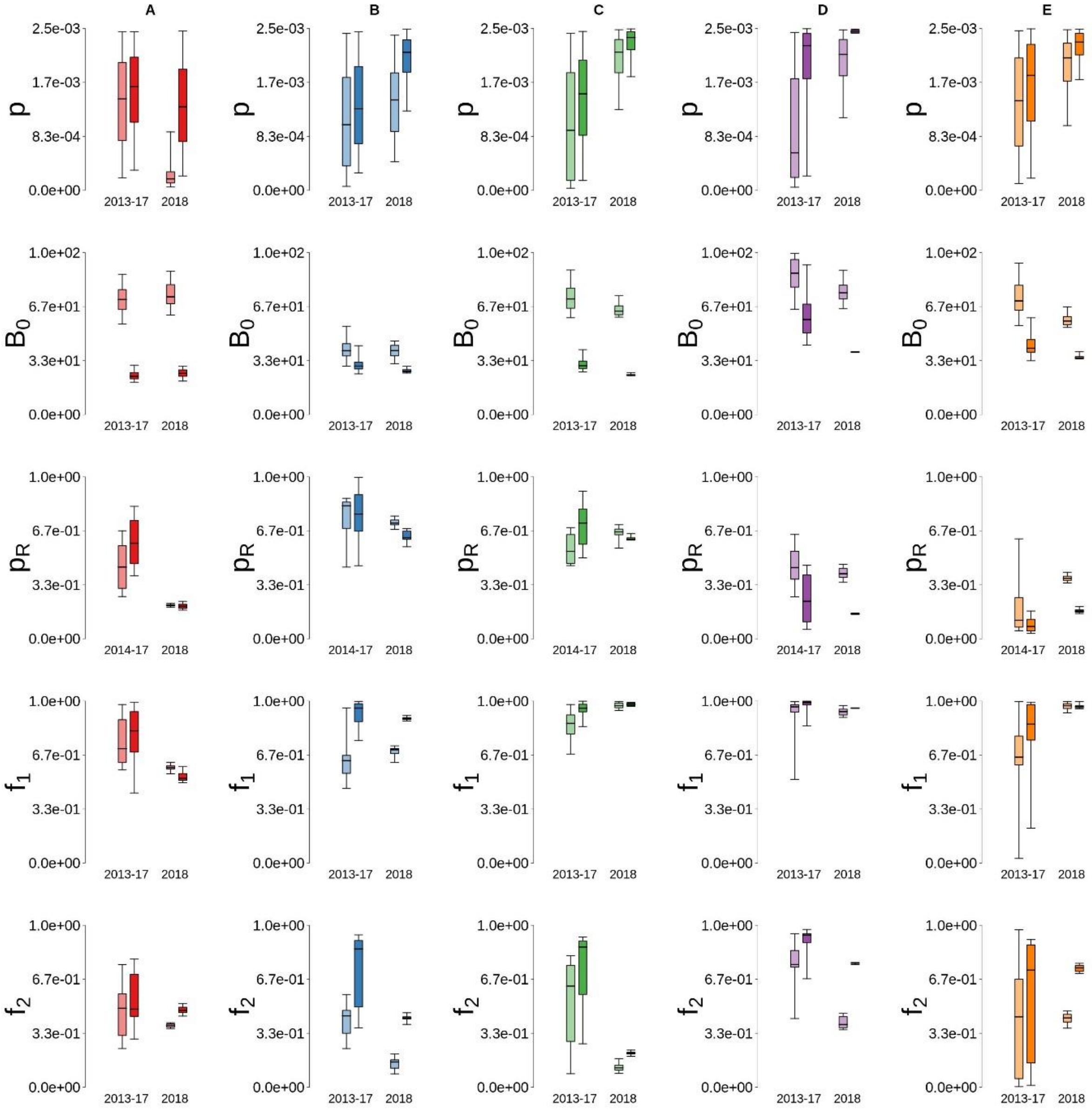

Figure J. Boxplots (2.5%, 25%, 75% and 97.5% quantiles and median) of the estimated distributions of the free parameters of the epidemiological model: p, B_0, p_R, f_1, f_2 (from first to last row) for each cluster A, B, C, D, E (from first to last column). Lighter colors show baseline distributions (see Figure C) while darker colors show estimates obtained by assuming the avian population to consist of house sparrows. Distributions are shown aggregating years before 2018.

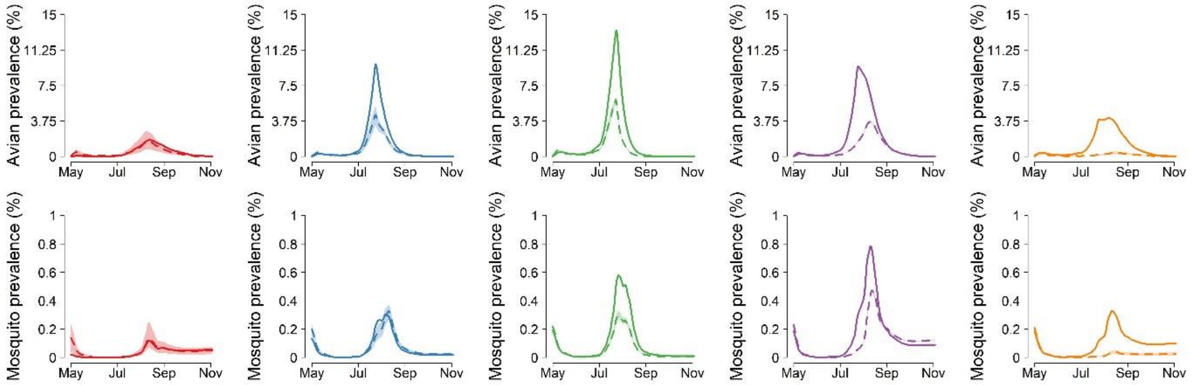

Figure K. Comparison between estimated average avian (first row) and mosquito (second row) prevalence with the baseline epidemiological model (continuous line) and by assuming the avian population to consist of house sparrows (dashed lines) for each cluster A, B, C, D, E (from first to last column). Shaded areas represent the 95%CI of simulations with house sparrows.

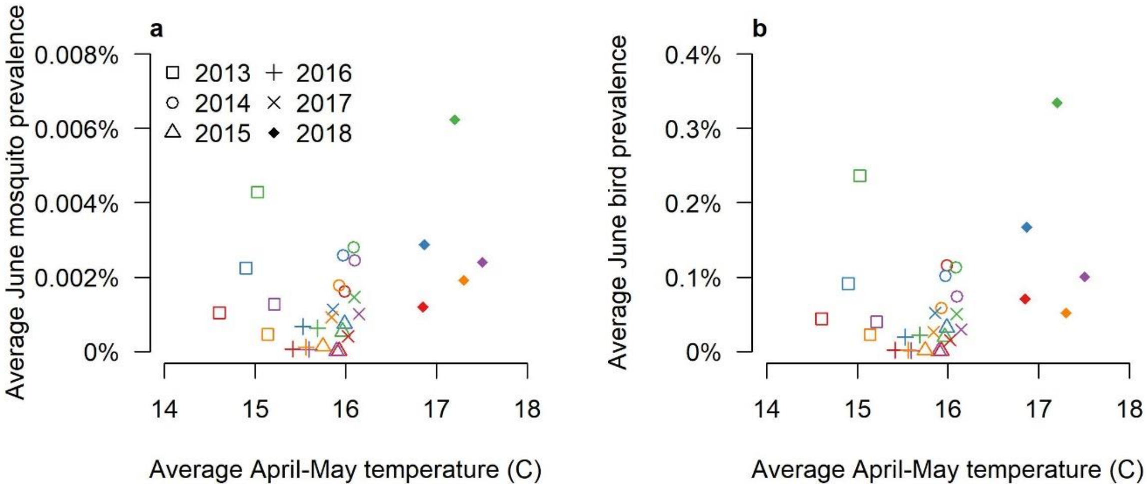

Figure L. Estimated average June mosquito (panel a) and avian (panel b) prevalence (Y-axis) versus spring average temperatures in degrees Celsius (X-axis) assuming the bird population to consist of house sparrows. Colors represent the cluster division (A: red, B: blue, C: green, D: purple, E: orange).

Human transmission

For each cluster $c \in \{A, B, C, D, E\}$ and day *t* of year $y \in [2013, 2018]$, we computed the human transmission risk $\lambda_H(c, y, t)$, similarly to what proposed in [5], as

$$\lambda_H(c,y,t) = \begin{cases} \frac{1-f_1}{d_A} \cdot \frac{M_i(c,y,t)}{h_c}, & t < \text{July } 15 \\ \frac{1-f_2}{d_A} \cdot \frac{M_i(c,y,t)}{h_c}, & t \geq \text{July } 15 \end{cases}$$

Where h_c is the average number of humans living in an area *a* for cluster *c* and $M_i(c, y, t)$ is the number of infected mosquitoes in *a* for cluster *c* at day *t* of year *y* as estimated by the model.

We then fitted the total number of WNND cases for each cluster *c* and year *y* following the same approach presented in [5]. In particular, we rescaled $\lambda_H(c, y, t)$ by a cluster-dependent parameter ρ_C, computed the total number of WNND cases predicted per year as

$$C_H(c,y) = \sum_t \text{Pois}(\rho_c \cdot \lambda_H(c,y,t) \cdot H_C)$$

Where H_c is the number of humans living in cluster *c*, and estimated ρ_C by Markov Chain Monte Carlo sampling applied to the Poisson likelihood of observing the recorded number of cases over the six years under study, given the model-predicted ones. We can interpret ρ_c as a product of the probability of virus transmission to humans per mosquito infectious bite times the probability of symptoms development times the reporting rate.

As presented in Figure M, 24 out of 30 (80%) observed yearly number of WNND cases lie within the 95%CI predictions of the human transmission model. The transmission parameter ρ_c is estimated to be significantly higher for cluster E (panel f), possibly implying quite different ecological settings with respect to the rest of the region. The majority of cases, in 2018, is predicted to occur between the end of July and the end of August (see Figure N), consistently to actual observations [10].

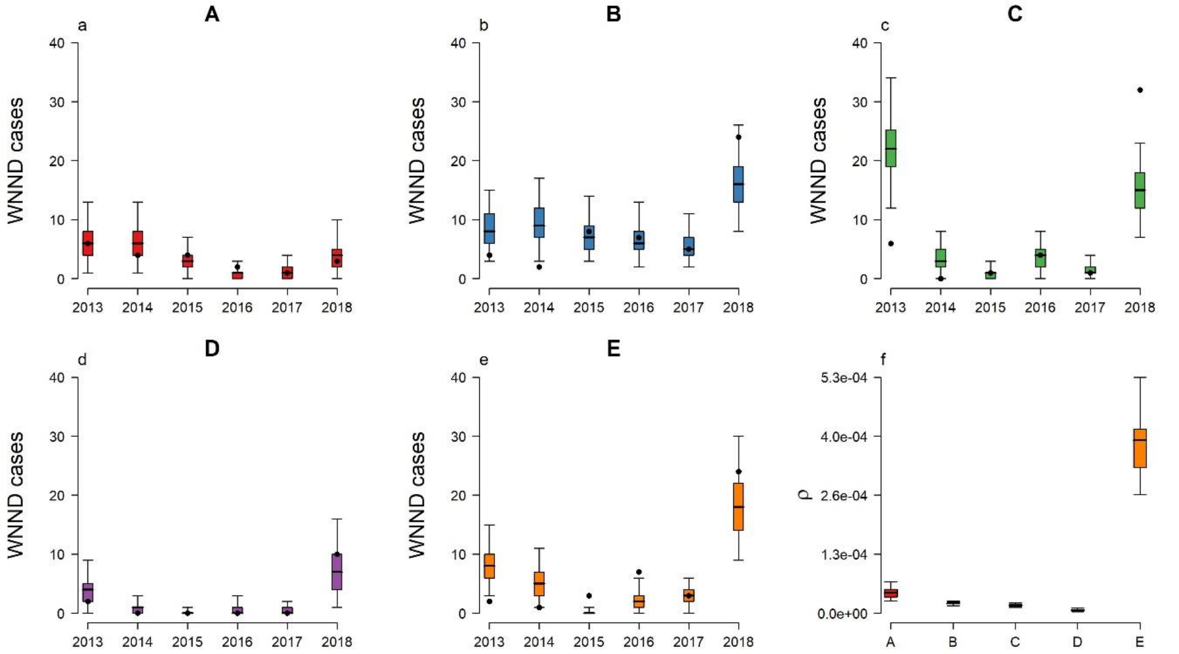

Figure M. Human transmission model results. a-e): Boxplots (2.5%, 25%, 75% and 97.5% quantiles and median) representing the distributions of the estimated number of WNND cases per cluster and year. Dots: observed data. f): estimated ρ_c distributions (2.5%, 25%, 75% and 97.5% quantiles and median) for the five clusters.

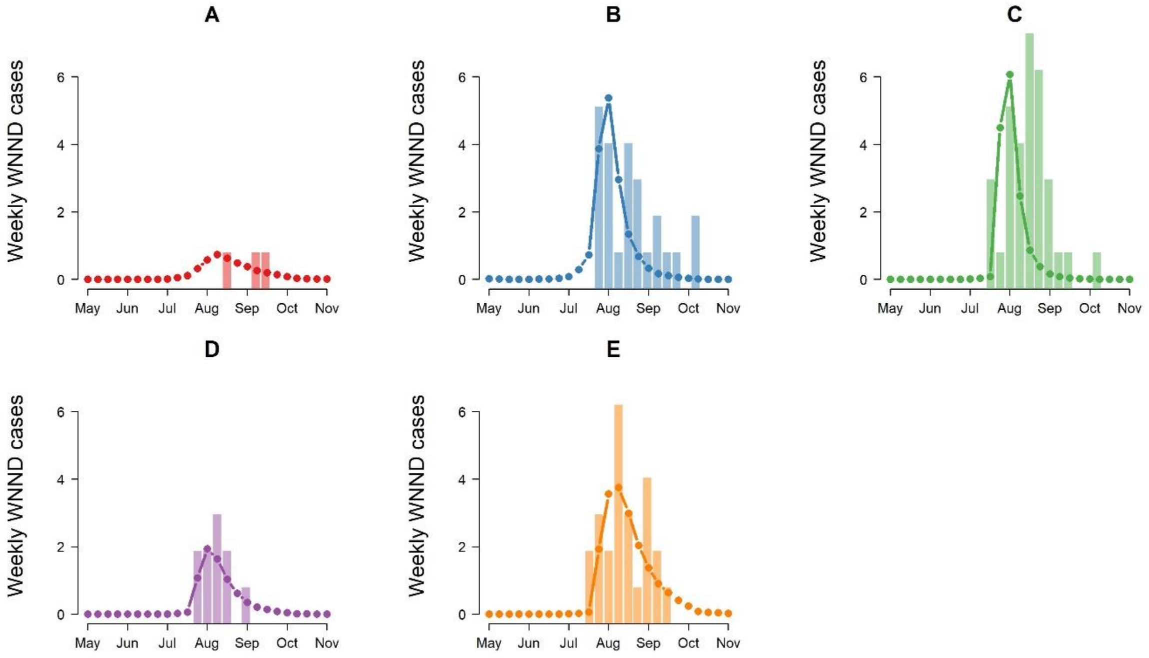

Figure N. Estimated average number of predicted (lines) and recorded (bars) WNND weekly cases per cluster for 2018.

References

1. Antolini G, Auteri L, Pavan V, Tomei F, Tomozeiu R, Marletto V. A daily high-resolution gridded climatic data set for Emilia-Romagna, Italy, during 1961–2010. Int J Climatol. 2016;36:1970–86.
2. Regione Emilia Romagna. Agenzia regionale per la prevenzione, l'ambiente e l'energia dell´Emilia-Romagna [Internet]. Available from: https://www.arpae.it/
3. Marini G, Poletti P, Giacobini M, Pugliese A, Merler S, Rosà R. The Role of Climatic and Density Dependent Factors in Shaping Mosquito Population Dynamics: The Case of Culex pipiens in Northwestern Italy. PLOS ONE. 2016;11:e0154018.
4. Marini G, Guzzetta G, Baldacchino F, Arnoldi D, Montarsi F, Capelli G, et al. The effect of interspecific competition on the temporal dynamics of Aedes albopictus and Culex pipiens. Parasit Vectors. 2017;10:102.
5. Marini G, Rosà R, Pugliese A, Rizzoli A, Rizzo C, Russo F, et al. West Nile virus transmission and human infection risk in Veneto (Italy): a modelling analysis. Sci Rep. 2018;8:14005.
6. Vogels CBF, Fros JJ, Göertz GP, Pijlman GP, Koenraadt CJM. Vector competence of northern European Culex pipiens biotypes and hybrids for West Nile virus is differentially affected by temperature. Parasit Vectors. 2016;9:393.
7. Bellini R, Calzolari M, Mattivi A, Tamba M, Angelini P, Bonilauri P, et al. The experience of West Nile virus integrated surveillance system in the Emilia-Romagna region: five years of implementation, Italy, 2009 to 2013. Eurosurveillance. 2014;19:20953.
8. Del Amo J, Llorente F, Figuerola J, Soriguer RC, Moreno AM, Cordioli P, et al. Experimental infection of house sparrows (Passer domesticus) with West Nile virus isolates of Euro-Mediterranean and North American origins. Vet Res. 2014;45:33.
9. Summers-Smith JD. The Sparrows: a study of the genus Passer. T & AD Poyser; 1988.
10. Istituto Superiore di Sanità (ISS). West Nile News. Aggiornamento epidemiologico settimanale, 2018 [Internet]. Available from: https://www.epicentro.iss.it/westNile/bollettino/Bollettino%20WND_%20N.%2018%20%2015.%2011%202018.pdf
